# Fast and accurate localization of multiple RF markers for tracking in MRI-guided interventions

**DOI:** 10.1007/s10334-014-0446-3

**Published:** 2014-05-07

**Authors:** Francesca Galassi, Djordje Brujic, Marc Rea, Nicholas Lambert, Nandita Desouza, Mihailo Ristic

**Affiliations:** Mechanical Engineering Department, Imperial College London, London, UK

**Keywords:** Magnetic resonance imaging, Tracking, MRI-guided intervention, 1D projections, RF markers

## Abstract

**Object:**

A new method for 3D localization of *N* fiducial markers from 1D projections is presented and analysed. It applies to semi-active markers and active markers using a single receiver channel.

**Materials and methods:**

The novel algorithm computes candidate points using peaks in three optimally selected projections and removes fictitious points by verifying detected peaks in additional projections. Computational complexity was significantly reduced by avoiding cluster analysis, while higher accuracy was achieved by using optimal projections and by applying Gaussian interpolation in peak detection. Computational time, accuracy and robustness were analysed through Monte Carlo simulations and experiments. The method was employed in a prototype MRI guided prostate biopsy system and used in preclinical experiments.

**Results:**

The computational time for 6 markers was better than 2 ms, *an* improvement of up to 100 times, compared to the method by Flask et al. (J Magn Reson Imaging 14(5):617–627, 2001). Experimental maximum localization error was lower than 0.3 mm; standard deviation was 0.06 mm. Targeting error was about 1 mm. Tracking update rate was about 10 Hz.

**Conclusion:**

The proposed method is particularly suitable in systems requiring any of the following: high frame rate, tracking of three or more markers, data filtering or interleaving.

## Introduction

Magnetic resonance imaging is increasingly being applied to perform interventional procedures such as biopsies, laser induced interstitial thermotherapy, high-intensity focused ultrasound and endoscopy [2–8]. In many procedures it is essential to be able to track the position of a device within the imaging volume of the scanner and to communicate this information in the form of an image feedback to the interventional staff. As the instrument is not visible in the MR image, fiducial markers mounted on the instrument are often used for its localization. Fast and robust localization of the fiducial markers is very important as it enables higher frame update rate that, in turn, makes imaging more consistent and, as such, improves hand–eye coordination. In this manner the errors introduced by the movement of both the patient and the interventional device are reduced [9]. Importantly, more accurate and faster targeting enables faster interventional procedures and decreases the cost of intervention [10].

Fiducial markers may be classified as active, semi-active or passive, each involving different localization techniques [11–14]. An active fiducial marker is a microcoil receiver, which provides the MR signal to a dedicated channel of the MRI system [11]. As the received signal is highly localized, this has the advantage of avoiding problems due to background noise. Multiple markers may be easily distinguished if each is assigned to a dedicated receive channel. A semi-active marker may be constructed as a resonant microcircuit, which is inductively coupled to a receiver coil of the MRI system [15]. Finally, a passive marker consists of an encapsulated volume of material chosen to provide good contrast in the acquired slices.

Both active and semi-active markers may be localized using 1D projections, by determining the location of the signal peak, while passive markers demand the use of image processing techniques, for example, template matching [13]. The use of 1D projections lends itself to much faster high-resolution data acquisition and processing than image processing of multiple slices [1]. Flask et al. [1] proposed a method for tracking *N* semi-active markers in 3D using 1D projections in two orthogonal planes and cluster analysis. Their algorithm requires at least five 1D projections per plane. However, projections with less than *N* peaks are rejected, so new projections are acquired instead. This results in an acquisition time of about 170 ms.

We propose a novel method for fast and accurate localization of *N* markers using 1D projections [16]. The method may be applied to localize either semi-active markers or active ones when only one receiver channel is used. By using more than three markers, higher accuracy in localizing an instrument may be achieved with no compromise in the update rate. The complexity of the post-processing algorithm was significantly reduced by avoiding cluster analysis [1], while high accuracy was achieved by using optimal projections to compute the points and by applying Gaussian interpolation in peak detection. The algorithm does not reject projections with coincident peaks, resulting in reduced scanning time, while the number of projections may be traded against robustness and accuracy, for optimized results in a specific situation. Performance has been characterized through a combination of extensive experimental studies and Monte Carlo simulations. The experiments involved wireless markers, a customized GRE sequence, and an MR-compatible moving platform. Preclinical volunteer studies were conducted to verify the method under patient loading and in the presence of physiological movements.

The proposed localization method was implemented within a custom made system for image guided prostate biopsy [17]. The application that integrates the tracking algorithm with a novel visualization method, specific for prostate biopsy, was implemented using Matlab and runs on a dedicated navigation workstation located in the control room and connected to the scanner via a local area network. The image reconstruction pipeline was programmed to stream 1D projections from the scanner to the navigation workstation just after the Inverse Fourier Transformation has been completed.

## Materials and methods

### Algorithm for localizing *N* markers from multiple 1D projections

#### Problem statement

**Fig. 1:**
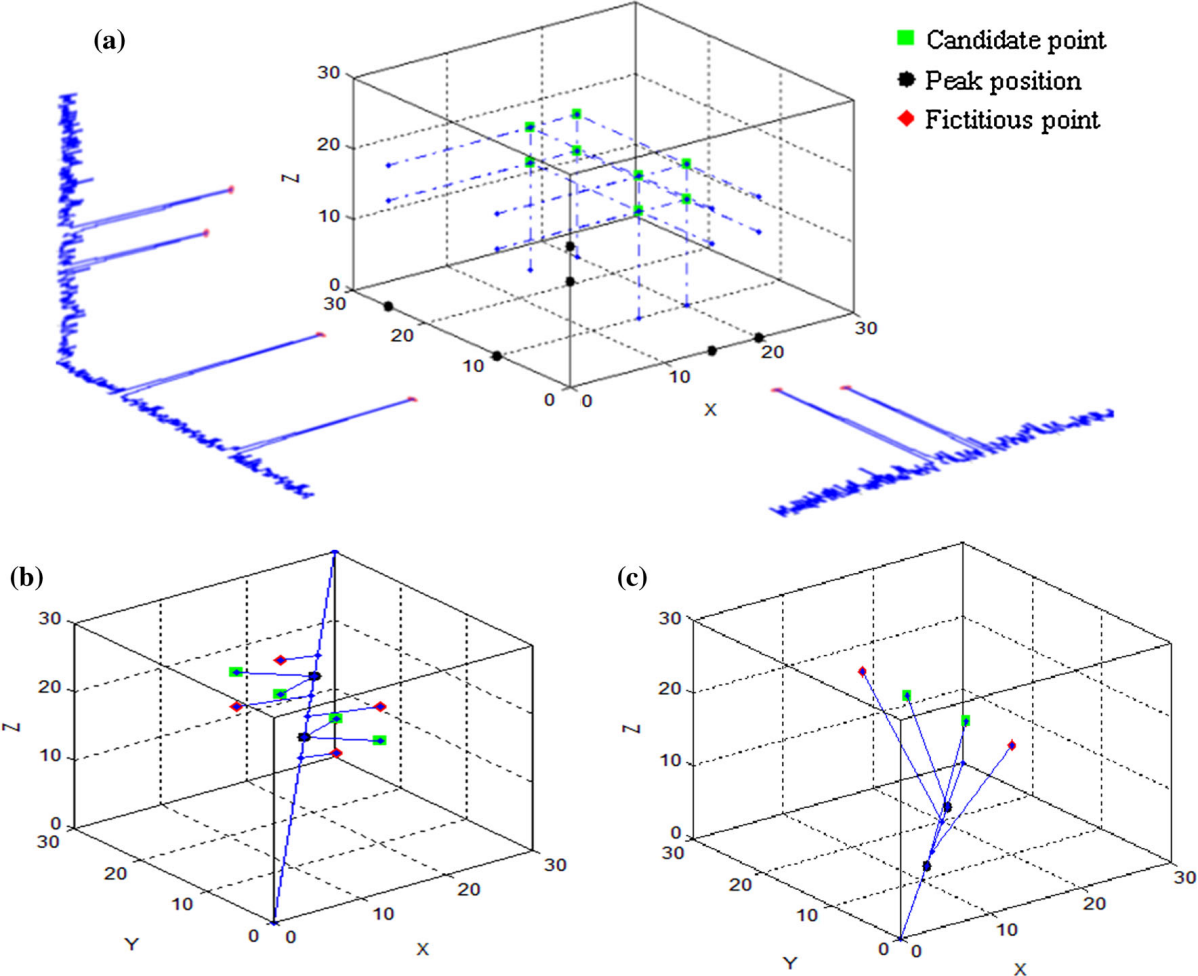
The algorithm for *N* = 2 markers. $N^{3} = 8$ intersection points are computed as candidates (**a**); by using a fourth projection four fictitious points are eliminated (**b**) and by using a fifth projection (**c**) only the true points are left

The* N* markers generate *N* or fewer peaks along a 1D projection. The problem is to compute the 3D coordinates of *N* markers by using *n* 1D-projections. Each detected peak defines a plane perpendicular to the corresponding projection direction. In general, three 1D projections of *N* points define three *N* planes which intersect at *N* ^3^ points. As a result *N* ^3 ^– *N* points are fictitious and must be discarded. This can be done by using additional projections (Fig. 1). Each candidate marker position is defined by the intersection of three planes, whose normals are not co-planar and intersect at a point ***P*** [18]:

1 $$\varvec{P} = \frac{{p_{1} \left( {\varvec{N}_{2} \times \varvec{N}_{3} } \right) + p_{2} \left( {\varvec{N}_{3} \times \varvec{N}_{1} } \right) + p_{3} \left( {\varvec{N}_{1} \times \varvec{N}_{2} } \right)}}{{\det \left( {\varvec{N}_{1} ,\varvec{N}_{2} ,\varvec{N}_{3} } \right)}},$$

where ***N***_*k*_ (*k* = 1, 2, 3) is the unit vector of the direction of a projection and *p*_k_ is the position of the peak along this direction.

#### Tracking algorithm

The method for tracking *N* markers consists of a number of steps, which are summarized below and further details are provided in the subsequent sections.

1. The process starts with the acquisition of a predefined set of 1D projections, involving excitation of the whole imaging volume. The number and directions of these 1D projections have been optimized, as presented below.
2. Peak detection is performed for each projection, the position of each peak is then determined with sub-pixel resolution.

Three *reference projections* are selected, as explained below, and *N* ^3^ candidate marker positions are calculated (Fig. 1a). The remaining projections are sorted according to the decreasing minimal distance between the peaks they contain. Projections with fewer than *N* peaks are placed at the bottom of the list and used last. In this way projections that may lose a peak have lower probability of being used and affecting the result. Note that projections with a smaller number of peaks, due to peak merging, will not eliminate any of the correct points.

The fictitious (N^3^ – *N*) candidates are eliminated by using, in turn, the remaining test projections. For each test projection, the projected value of the each candidate point is calculated and the distances between this and the identified peak locations are computed (Fig. 1b, c). The candidate is removed if the projected point does not have an identified peak in its vicinity, i.e., if the minimum computed distance is larger than an experimentally determined tolerance *ɛ*. If the number of computed points is different from the known number of markers, then the entire solution is discarded.

#### Choice of the 1D projection directions

Each computed peak location $p_{k}$ has an error $\Delta p_{k}$ associated with the measurement. The errors $\Delta p_{k}$ result in an error $\Delta \varvec{P}$ in the computed point $\varvec{P}$, thus,

2 $$\varvec{P} = \varvec{P}_{0} + \Delta \varvec{P},$$

where $\varvec{P}_{0}$ is the true position of the marker. It follows from (1) and (2) that

3 $$\Delta \varvec{P} = \frac{{\Delta p_{1} \left( {\varvec{N}_{2} \times \varvec{N}_{3} } \right) + \Delta p_{2} \left( {\varvec{N}_{3} \times \varvec{N}_{1} } \right) + \Delta p_{3} \left( {\varvec{N}_{1} \times \varvec{N}_{2} } \right)}}{{{ \det }\left( {\varvec{N}_{1} ,\varvec{N}_{2} ,\varvec{N}_{3} } \right)}}.$$

**Fig. 2:**
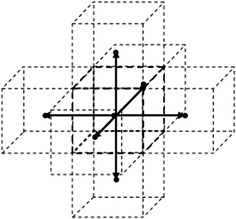
6-neighbourhood of a voxel. A voxel is connected to the six surrounding voxels which have a face in common

The error $\Delta \varvec{P}$ may be minimized by maximizing $\det \left( {\varvec{N}_{1} ,\varvec{N}_{2} ,\varvec{N}_{3} } \right)$. This is achieved by maximizing the minimum angle between any two directions. In other words, the projection directions should be regularly distributed in space. The direction candidates were taken from the voxel neighbourhood [19], a well-known concept in computer graphics. For a 6-neighbourhood of a voxel this is illustrated in Fig. 2, which defines three regularly-distributed projection lines. Similarly, 18-neighbourhood and 26-neighbourhood define, respectively, nine and 13 regularly distributed projection lines.

The number of projection directions needs to be known in advance in order to keep the tracking sequence simple and to achieve a high update rate of the localization method. Simulations were used in order to test the performance of the algorithm in relation to the number of projections. As a result, 13 projections were acquired and their directions defined in accordance with the 26-neighbourhood.

#### Selection of the reference projections

Following the acquisition, peak detection and peak localization, three reference projections need to be selected from the acquired set. For the case of 13 acquired projections there are $13!/\left( {13 - 3} \right)!3! = 286$ subsets of three directions $\varvec{N}_{i} ,\varvec{N}_{j} ,\varvec{N}_{k}$. The subsets are ordered according to the decreasing value of the determinant $\det \left( {\varvec{N}_{1} ,\varvec{N}_{2} ,\varvec{N}_{3} } \right).$ The first subset with *N* distinct peaks and a minimum distance between two peaks greater than a prescribed value *d* is selected as the reference subset. The latter condition ensures that the candidate points are well distributed in the volume so that the corresponding computed projected values are well distributed along a projection direction, which improves the success rate in the subsequent removal of fictitious points.

### Fiducial markers and tracking sequence

#### Fiducial markers

**Fig. 3:**
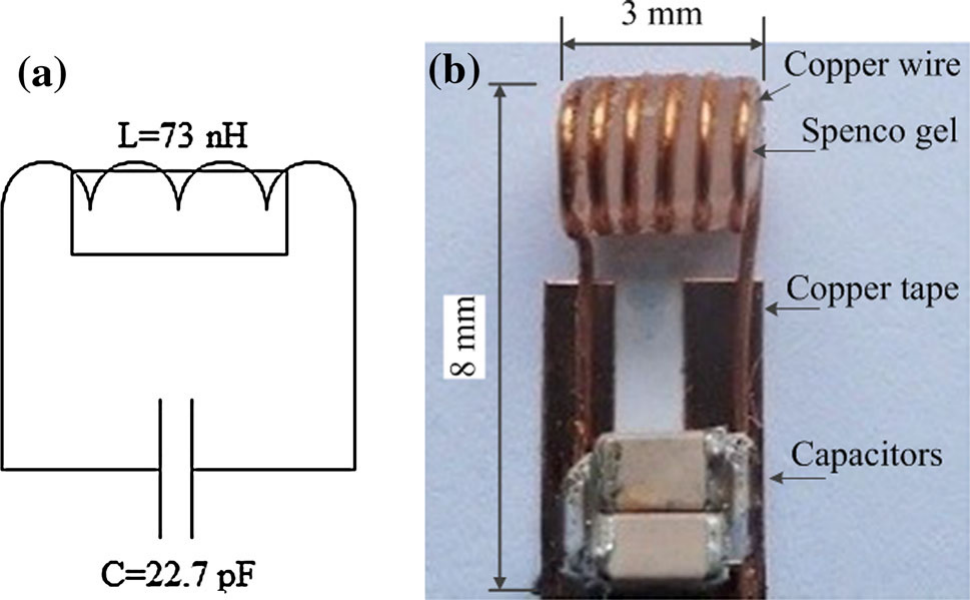
Wireless RF markers. Circuit (**a**) and marker (**b**), tuned to the frequency (123.5 ± 0.05) MHz. The marker comprises two non-magnetic capacitors and a 3 mm diameter inductor, filled with water–gel (relaxation times T_1_ = 160 ms and T_2_ = 14 ms) and soldered on two copper tape strips. The inductor was sealed with glue to avoid contamination of the water–gel and for mechanical rigidity

Fiducial markers were constructed similarly to [20], consisting of 3 mm diameter wireless micro-coils tuned to the Larmor frequency of the scanner and filled with high ^1^H density, water-gel material (vinyl plastisol gel, Spenco Healthcare, Horsham, UK) (Fig. 3).

A Vector Network Analyzer (Anritsu MS 2026A VNA Master) was used to fine tune the RF marker to $$f_{r} = 123.5\,{\text{MHz}}$$ by using inductively coupled test coils connected to the reflection and the transmission channel, respectively. By measuring the bandwidth $$\Delta f$$ at −3 dB between lower and upper cut-off frequency, the quality factor was calculated as $${\raise0.7ex\hbox{${f_{r} }$} \!\mathord{\left/ {\vphantom {{f_{r} } {\Delta f}}}\right.\kern-0pt} \!\lower0.7ex\hbox{${\Delta f}$}}$$.

#### Tracking sequence

A 2D spoiled GRE sequence was modified in order to acquire a predefined set of 1D-projections, each projection including a dephasing gradient to spoil the transverse component of the magnetization prior to the next acquisition. The following parameters were made adjustable from the user interface of the MR host computer: (1) flip-angle, (2) magnitude of the dephaser gradient and (3) time interval between individual projections. Other relevant sequence parameters were TR = 5.6 ms, TE = 3.5 ms, FOV 300 mm, 320 phase encode points.

#### Safety assessment

The potential local heating due to electromagnetic coupling to the RF transmitter is an important issue to be considered when using RF semi-active markers in the clinical environment [21]. In order to verify the thermal safety of the markers, an optic fibre thermometer (Luxtron 812, LumaSense) with the accuracy of 0.1 °C was used. Similarly to [21], the optic fibre sensor was taped directly onto a marker which was, in turn, placed on top of a large water phantom. The test was performed in a 1.5T MR Siemens scanner. The temperature was recorded over 11 min, with no RF excitation during the first minute (base line) and RF excitation present during the subsequent 10 min. The test was performed for a T2-weighted turbo spin echo (TSE) sequence (whole body specific absorption rate SAR = 0.7 W/kg), which is the imaging sequence routinely used in prostate imaging, and for the developed tracking sequence (SAR < 0.001 W/kg). The SAR values were reported by the scanner. In both experiments the body loading was represented by two 2 l phantoms placed adjacently near the scanner’s isocentre. TSE imaging sequence parameters (TR = 3,670, TE = 121 ms, flip angle = 137°, slice thickness 3 mm, distance factor 10 %, base resolution 320, phase resolution 70 %, turbo factor = 23) were set according to the protocol for prostate imaging at Royal Marsden Hospital (Sutton, London, UK).

### Signal analysis

#### Signal amplitude under repeated excitation

The micro-coil locally amplifies the excitation field. For an applied flip angle $\alpha_{\text{app}}$ it results in an effective flip angle $\alpha_{\text{eff}} = Q \times \alpha_{\text{app}} ,$ where *Q* is the *Q*-factor of the micro-coil [15]. With $TR \ll T_{1}$, the magnetisation is expected to approach the steady state over a set of projections [22]. The aim is to maximize the amplitude of the received MR signal for each projection, therefore to maximize the transverse magnetization component at the echo time TE. Similarly to Hargreaves et al. [23], the magnetization vector was derived as:

4 $$M_{\text{TE}} = AM_{i} + B,$$

where *M*_*i*_ is the magnetization vector just before the RF pulse, A and B are matrices representing the RF nutation about the *x*-axis, the precession about *z*-axis and the T_1_ and T_2_ relaxation processes. The transverse component $M_{{{\text{TE}}_{\text{XY}} }}$was then computed as $M_{{{\text{TE}}_{XY} }} = \sqrt {M_{{{\text{TE}}_{X} }}^{2} + M_{{{\text{TE}}_{Y} }}^{2} }$ and simulated (T_1_ = 160 ms, T_2_ = 14 ms, TE = 3.5 ms, TR = 5.6 ms) for a marker under repeated excitation and for flip angles $0.1^\circ < \alpha_{\text{app}} < 0.5^\circ$. The flip angle *α*_app,_ which gives higher amplitude of the received signal, was thus estimated. Experimentally, 1D projections were acquired in the presence of the large water phantoms.

#### Marker orientation to the main magnetic field

Orientation of the marker to the main field is an important factor to be considered when designing interventional devices [15]. At some angles the amplitude of the signal might be comparable with the background signal and; hence, peak detection may become unreliable. This was investigated experimentally using a custom made rotary holder to position the marker’s axis at various known angles to the main field. The induced RF flux is dependent on the angle between the axis of the coil and B_1_ field. The signal is in turn dependent on induced RF flux and its orientation with respect to the main field. These dependencies tend to reduce the signal as the coil axis rotates from B_1_ to B_0_. In our experiment a marker was placed at the scanner’s isocentre, on top of the water phantom and rotated in steps of 10^∘^ in the xz plane. At each step, 20 measurements were acquired and the average amplitude of a peak was calculated and compared to the background signal.

#### Peak detection and sub-pixel localization

Inverse Fourier transform of a 1D projection produces a signal with peaks that correspond to the positions of the markers. Correctly identifying the peak is essential for the success of the algorithm. The presence of background noise in the acquired MR signal makes simple thresholding inadequate for robust peak detection. Following the method suggested in [1], peak detection starts with a search through the sequence of values in 1D projection to identify local maxima using discrete differentiation. The *N* largest peaks are checked against an experimentally determined signal to noise threshold, *λ*. The value of *SNR* is calculated by dividing the magnitude of each peak by the standard deviation of the background noise [1] computed after removing each of the *N* peaks and their adjacent four points on either side. Only the peaks satisfying the threshold are accepted.

The location of the peak is corrected by applying an algorithm for sub-pixel peak detection. In order to achieve sub-pixel accuracy we have used Gaussian interpolation as suggested in [24]. Gaussian interpolation uses signal amplitude value *b* at the location of the highest signal value, *x*, and the signal values *a* and *c* at the adjacent positions on the left and on the right side of it, respectively [24]:

5 $$\hat{X} = x - \frac{1}{2}\left( {\frac{\ln \left( c \right) - \ln \left( a \right)}{\ln \left( a \right) + \ln \left( c \right) - 2\ln \left( b \right)}} \right).$$

The variation in the location of a peak over repeated acquisitions, with the marker in the same position, was explored experimentally. This was considered essential for the assessment of the accuracy of the localization. A marker was placed at eight different distances from the isocentre, and for each position 20 repeated sets of 1D-projections were acquired. Peak localization was performed using the sub-pixel peak detection algorithm. For each projection, the mean position of a peak and the deviations from the mean were computed.

 A Chi square goodness-of-fit test of the null hypothesis that these variations are from a normal distribution was performed over repeated acquisitions of 1D projections of a marker. The hypothesis was verified and, consequently, a normally distributed variation in the position of a peak was implemented in Monte Carlo simulations of the algorithm.

The influence of the SNR on the accuracy of the sub-pixel estimation was explored through simulations. Three points that define the maximum were obtained from the experimental data. Realistic noise was added to the amplitude of all three points and its effect on the estimated peak position was studied.

#### Peak merging

Two markers which have the same coordinate along a gradient direction induce signals at a similar frequency [25]. In this situation, the normally separate multiple peaks may be at the limit of the spectral resolution of the system, and; hence, their peaks may merge in some acquisitions and not in others. This was investigated and the results were used to determine the tolerance *ε*.

### Performance assessment

A large number of simulations and experiments were performed in order to verify the localization method, to determine the required number of projections and to assess the performance in terms of markers localization accuracy, targeting accuracy, robustness and computational time.

In addition, the localization method was tested as an integral part of the MRI-guided prostate biopsy system that is currently under development in our research group [17, 26, 27]. Preclinical tests involving healthy volunteer subjects were conducted in order to verify successful localization by the proposed method under normal loading conditions and in the presence of physiological movement.

#### Simulations

Configurations of up to six markers used for the probe localisation were studied. Monte Carlo simulations were performed in order to establish the required number of projections, to assess the accuracy and robustness of 3D localization and to estimate the computational time. For each number of markers *N*, 10^5^ sets of *N* points in 3D were generated with the only constraint that they were as least 30 mm apart. Each set of *N* markers positions was projected onto all projection directions and noise derived from the Gaussian distribution was added to the projections. These values provided the input to the tracking algorithm. The statistical analysis was performed by varying the number of simulated fiducial markers from three to six and the number of projection directions employed from five to 13.

The proposed localization method was compared to the method proposed by Flask et al. [1], which we also implemented in Matlab and tested on the same computer (Windows PC, i7 processor 2.13 GHz, 4 Gb RAM).

In addition to the marker localisation error, we have analysed the targeting error in a situation when multiple markers are used to track an interventional device within the scanner imaging volume. Targeting error was defined as the distance between corresponding points other than the fiducial points [28]. Figure 5a indicates positions of three markers on an endorectal prostate biopsy probe. For analysis purposes, realistic marker configurations involving three, four, five and six markers were chosen. Monte Carlo simulation was performed for each configuration, involving 10^5^ random probe rotations in the range 30°–80° in the sagittal plane and ±25° in the coronal plane. For each probe orientation, the positions of the markers and their projections were computed. Noise derived from the Gaussian distribution was added to the projections. The markers were reconstructed from projections, assigned to the model markers and aligned in least squares fashion with the corresponding nominal points. The position of the biopsy needle tip was computed from known geometry and targeting error was computed as the distance between the computed and the nominal needle tip position.

#### Experiments

**Fig. 4:**
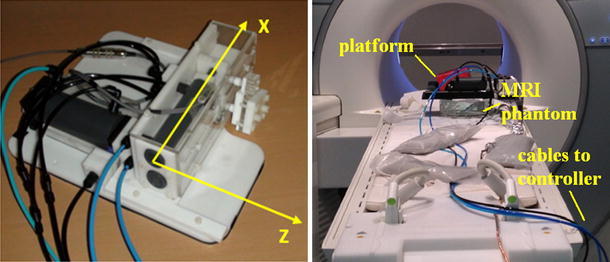
MRI-compatible pneumatic moving platform controlled remotely from control room

Experimental accuracy assessment employed a 2 degree-of-freedom, pneumatic, remotely controlled, MR-compatible platform, developed by our group (Fig. 4). The platform incorporates incremental, linear optical encoders that provided independent positional measurements with the resolution of 0.025 mm. Both static and dynamic tests were performed. In all performance assessment experiments, a body coil was used as well as large water phantoms (two bottles of 1,900 ml each, solutes per 1000 g H2O dist.: 3.75 g NiSO4·6 H2O + 5 g NaCl, placed adjacently in the vertical position, near the iso-centre).

##### Static experiments

A marker was fixed on the moving arm of the platform (Fig. 4) in order to assess the accuracy of its localization. The platform axes were aligned with the scanner axes and the marker was placed at the scanner’s isocentre by means of the laser crosshair of the scanner. Translations were performed in 10 mm steps along *x* and *z* axes in the range ±40 mm. At each step the marker’s position was measured 20 times by the proposed method and once by the platform’s optical encoders. Standard deviation and maximum distance between the computed positions and the mean value were calculated. The differences between the distances computed using the proposed algorithm and those measured using optical encoders were analyzed.

The accuracy of marker localization was also assessed at various positions in the *xy* plane. A marker was mounted on a custom-made rotary holder, placed beside a water phantom, rotated through five positions in the range 0–90° and scanned 20 times in each position. A circle was fitted to the resulting 100 points and the distances of the estimated positions from the circle were computed.

For the fitted circle *R* ^2^, goodness of fit was computed [29]:

6 $$R^{2} = 1 - \frac{{\sum\nolimits_{i = 1}^{n} {\left( {y_{i} - f_{i} } \right)^{2} } }}{{\left( {y_{i} - y_{\text{av}} } \right)^{2} }},$$

where $f_{i}$ is the predicted value from the fit and *y*_av_ is the mean of the observed data *y*_*i*_.

##### Dynamic experiments

The assessment of the accuracy under dynamic conditions necessitated the use of independent real-time position measurement that could not be provided with a volunteer present in the scanner. The moving platform was, therefore, used for this purpose in combination with a large water phantom. Dynamic tests involved moving a fiducial marker along predefined trajectories in x and z directions at various speeds, while simultaneously recording the encoder readings and the estimated fiducial positions.

#### Application: MRI guided prostate biopsy

**Fig. 5:**
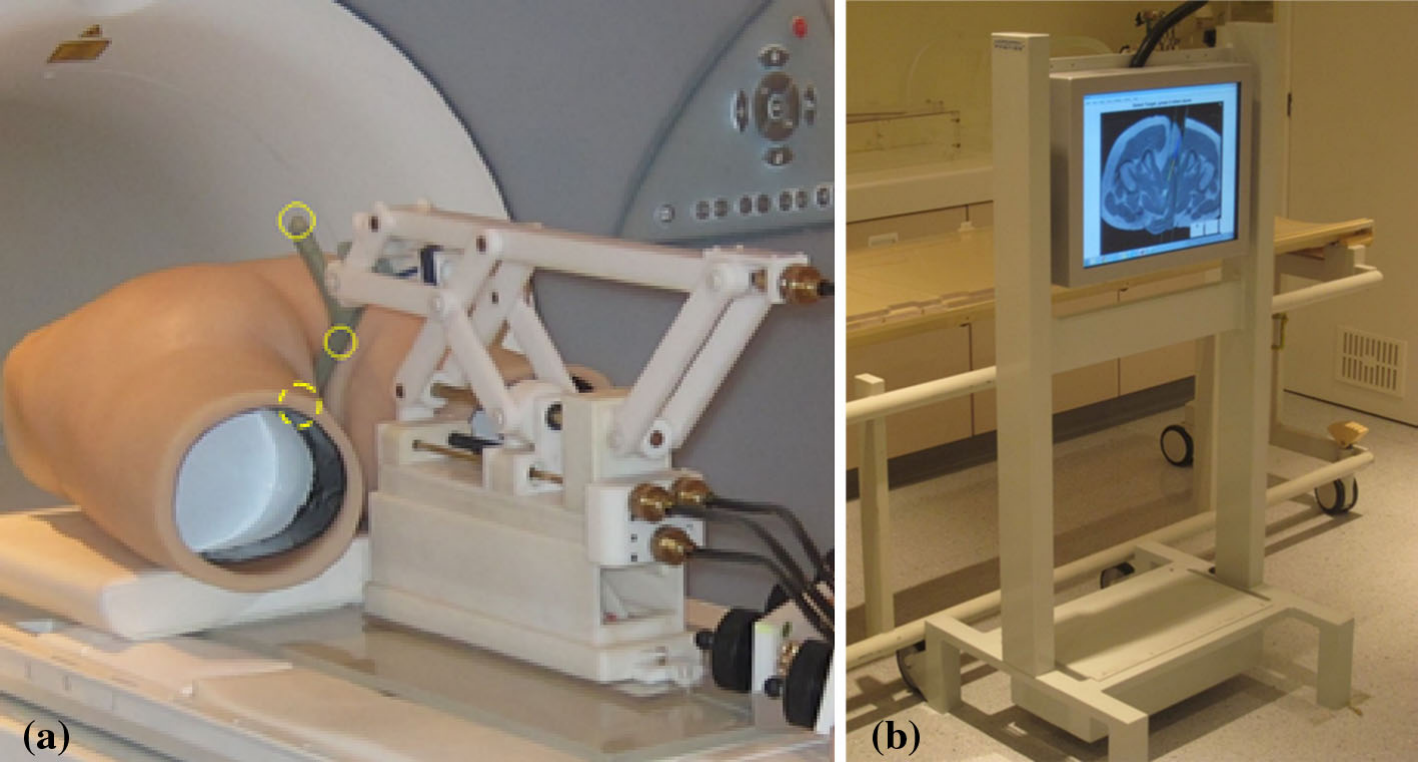
**a** MRI-guided prostate biopsy system; yellow circles indicate positions of the markers, **b** screen in a scanner room

The MRI-guided prostate biopsy system employs a remotely operated MRI-compatible manipulator used to position a detachable endorectal probe that serves as the biopsy needle guide. Figure 5a shows the set-up in the MR scanner. For the purposes of preclinical testing, we have also constructed an MRI-visible pelvic model made from silicon rubber, which realistically represents the male pelvic anatomy, both internally and externally, of a person weighing approximately 70 kg. The overall dimensions of the model were 50, 50, 20 cm. The model houses anus, rectum and an empty cavity for placement of a gelatine assembly which did possess the density and elasticity properties that mimic the resistance of normal prostate tissue and have a number of simulated lesions in it. The model provides realistic displacements of the lesion that are caused by the probe movement. In addition, the model accommodates the two large water phantoms described previously, which provided the loading. This setup was primarily used to assess the suitability of the manipulator for use in a limited space and to assess the clinician’s perception of the situation in a scanner.

The procedure starts by positioning the pelvic phantom onto the scanner bed and by inserting the probe. The manipulator is then fixed in place on the scanner bed and the probe is attached to it. A preoperative set of images is acquired and the scene is constructed using the transversal and interpolated coronal cross section through the lesion. The clinician identifies the lesion to be targeted, interactively selects the target point and starts the targeting. During the targeting, the scanner streams 1D projections to the navigation workstation, which computes the probe position. The 3D synthetic model of the probe is inserted into the scene in real time and the scene is visualized on the screen in the scanner room (Fig. 5b). To improve the clinician’s comprehension of the current situation in the scanner, the oblique cross section, passing through the markers, is interpolated through the scanned volume every time new marker positions are obtained. Note that the scanned volume, the scene and the probe positions are all computed in the scanner’s coordinate system. To make targeting easier, the needle is visualized in a position where it would be if fired. Based on the current position of the needle in relation to the selected target position, the clinician remotely controls the manipulator and moves the probe towards a correct firing position.

Before releasing the needle, a verification scan is performed in the same way as the preoperative one. The position of the targeted lesion is assessed and, if significant movement is detected, then the whole procedure is repeated until satisfactory targeting is achieved. At that point an automatic biopsy gun incorporating an extended, MRI compatible, biopsy needle (InVivo, Germany) is introduced into the device using an elongated handle. The needle is fired and the sample is acquired.

**Fig. 6:**
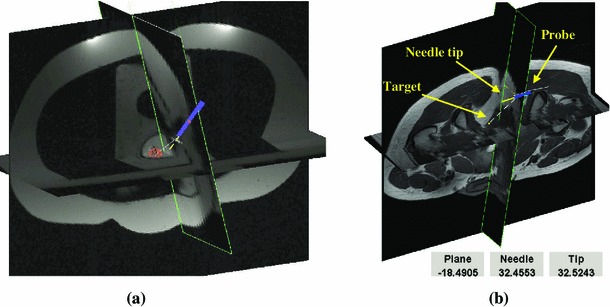
Intraoperative image guidance: **a** visual feedback during preclinical pelvic phantom trials; **b** volunteer scan used to illustrate how actual procedure would be carried out, showing the desired needle alignment (*dashed*) and relevant instantaneous distance measurements

Figure 6a shows the display during the preclinical phantom trials, while Fig. 6b overlays a 3D model of the probe onto the volunteer’s scan to show how this may look in a real situation.

Probe localization during the procedure is performed using markers built into the probe, Fig. 5a. Importantly, the manipulator mechanism was designed such that the axes of the markers are always kept perpendicular to the main field. In order to compute position and orientation of the needle, it is necessary to assign the points computed by the localization method to the corresponding ones on the nominal model of the probe. The assignment is based on the fact that, by design, the distances between the markers are different from one another, and they are known [25]. The marker that is common to the two largest distances is annotated as ‘A’, while the other one on the largest distance is annotated as ‘B’. The third marker is annotated as ‘C’. Probe localization is performed as a rigid body transformation that minimizes the sum of the squared distances between the computed points and the nominal ones [30]. Following this, the needle direction and the needle tip position are computed from the known geometry of the probe.

#### Volunteer experiments

Preclinical volunteer experiments were conducted in order to verify that the localization method performs successfully under the conditions when the patient is present in the scanner, where the main potential issue is the SNR. The experiments also aimed to verify that the localization method is successful in the presence of physiological movement. In addition, these studies provided an assessment of the setup time and other aspects needed to carry out the intended prostate biopsy procedure, although the tests were strictly non-invasive.

Volunteers were healthy men of the age 28, 40 and 53 and weighing 72, 76 and 90 kg, respectively. Volunteers were enrolled in an investigational protocol reviewed and approved by the institutional review board after providing the informed consent. In one set of experiments, a biopsy probe with embedded three markers was detached from the manipulator and held tightly between the subjects’ legs. The markers were continuously localized using the proposed method at ten updates per second over a period of several minutes. Images using the relevant TSE sequence or TSE imaging sequence were also obtained (TR = 3,670, TE = 121 ms, flip angle = 137, slice thickness 3 mm, distance factor 10 %, base resolution 320, phase resolution 70 %, turbo factor = 23). In other experiments the markers were attached to the volunteers’ anterior abdominal wall using adhesive tape in order to verify that the method is successful in the presence of respiratory movement. Continuous localization was performed at ten updates per second over a period of several minutes.

## Results

### Microcoil characteristics and safety

The Q-factor of the microcoil was found to be around 130 without loading, while when loaded by placing it on top of a water phantom the Q-factor was found to be around 120.

No measurable temperature change was observed during the experiments with tracking sequence. For the imaging sequence a temperature rise of about 0.2 °C was recorded.

### Signal analysis

#### Signal amplitude under repeated excitations

**Fig. 7:**
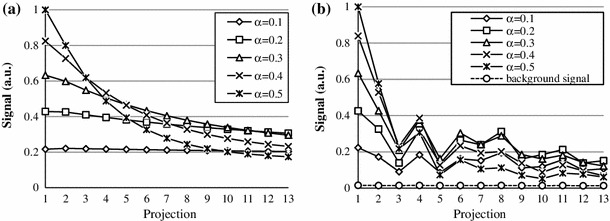
Signal variation under multiple excitations. **a** Simulated and **b** experimentally acquired signal for different flip angles

Figure 7 shows simulated and experimentally received signals, generated by the marker when acquiring a set of 13 projections at different applied flip angles $\alpha_{\text{app}}$. Simulations and experiments show that for higher flip angles *α*_app_, the amplitude of the acquired MR signal is initially larger; however, for the subsequent acquisitions, the amplitude drops faster than for smaller flip angles and reaches values comparable with the measured background signal. The flip angles *α*_app_ for which the signal showed higher minimum amplitude of signal over the whole set were 0.2° and 0.3°, the effective flip angle was $Q \times \alpha_{\text{app}}$.

The experimental signal shows an oscillating trend (Fig. 7b). This was found to be highly repeatable and dependent on the direction of projection, therefore, it was attributed to an asymmetry of the markers.

#### Marker orientation to the main magnetic field

**Fig. 8:**
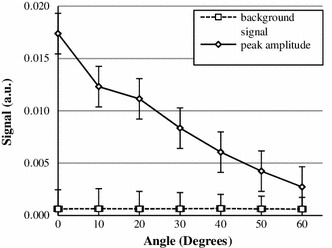
The amplitude of a peak decreases for increasing angles to the main magnetic field

Measured results in Fig. 8 show that for angles up to 50° peaks were always correctly detected for all the projections. At 60° the amplitude of the peak was comparable with the amplitude of the background signal and the peak was correctly detected in about 60 % of the cases.

#### Peak detection and sub-pixel localization

**Fig. 9:**
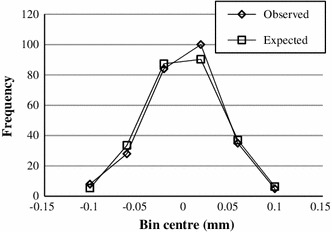
A number of events from the known distribution expected in each bin and a number of events observed in each bin

Using the position of the pixel with the highest intensity for peak localization, would lead to a maximum error of 0.5 pixel, or 0.6 mm in this work. By using Gaussian interpolation, the maximum peak localization error was reduced to 0.283 mm (Table 4). The Chi square goodness-of-fit test showed that the deviation of the peak positions over repeated acquisition is normally distributed (Fig. 9). The standard deviation $\sigma_{\text{peak}}$ varied between 0.03 and 0.075 mm. In the experiments with a volunteer in the scanner the standard deviation reached 0.08 mm.

#### Peak merging

**Fig. 10:**
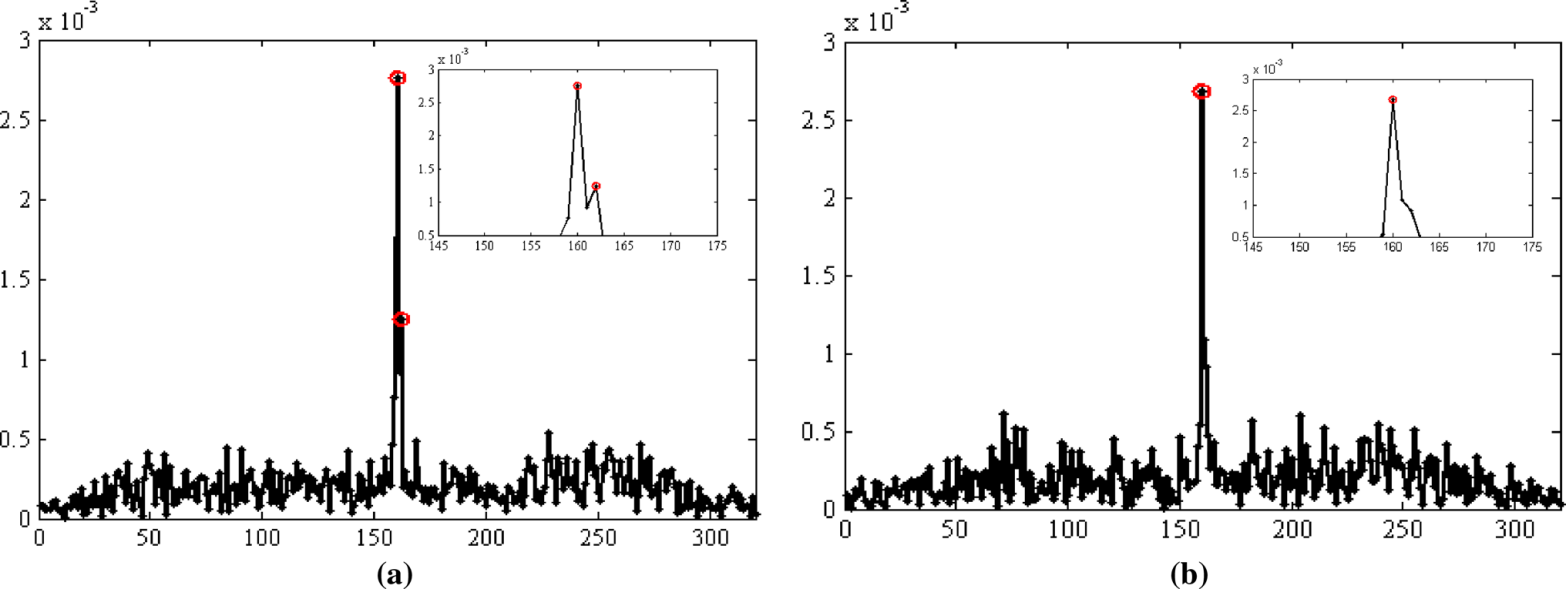
Identification of close peaks. **a** Two distinct peaks detected, **b** single peak detected

A situation involving two close peaks is illustrated in Fig. 10, where the same projection was acquired twice while the markers were static. In Fig. 10a two high intensity peaks are evident while in Fig. 10b these peaks have merged into one. It was found that peak merging may happen when peaks are within two pixels from one another and that the position of the identified peak is not affected by merging. This phenomenon, however, does not affect the accuracy in determining the candidate points, and, therefore, the accuracy of localization, because projections with less than *N* peaks are not used in their computation. The only consequence is that in situations with less than *N* peaks along some direction the tolerance needs to be enlarged to the size of two pixels.

It was also noted that a single marker may cause the occurrence of two distinct peaks in a single projection. In all such cases the distance between these two peaks was exactly one pixel. This can potentially cause serious problems as a fictitious peak may be higher than some real peak and, therefore, lead to entirely inaccurate conclusions. However, this was solved by a simple remedy: if two peaks are exactly one pixel apart, then the smaller of the two is neglected. In this way the problem becomes the same as the one explained above and, as such, it does not affect the accuracy.

One more effect has been identified that needs attention. If the density of the material within the coil is non-uniform, or, if the shape of the material within a coil is not spherical, the maximum signal intensity may not appear at the centre of the coil, depending on the orientation of the coil. This may affect the accuracy of localization—so every effort has been made to make the shape symmetric and the homogeneity of the material in the coil uniform, as much as possible.

### Performance assessment

#### Simulation studies

##### *Robustness and Accuracy*

The robustness of the proposed method was expressed in terms of a percentage of successful localizations of *N* markers in a set of experiments. Table 1 summarizes the results of 10^6^ simulations of *N* markers, with *N* = 3, 4, 5, 6. The number of 1D projections was increased from five up to 13. These results show that robustness is improved with an increased number of projections and that more projections are needed with an increased number of markers. In all cases simulated variation in peak localization was σ_peak_ = 0.08 mm.

**Table 1:** Percentage of successful marker localizations

	5 proj.	7 proj.	10 proj.	13 proj.
3 markers	95.40	99.99	100.00	100.00
4 markers	86.00	99.94	99.998	100.00
5 markers	70.00	99.83	99.994	99.9991
6 markers	51.00	99.40	99.98	99.999

**Fig. 11:**
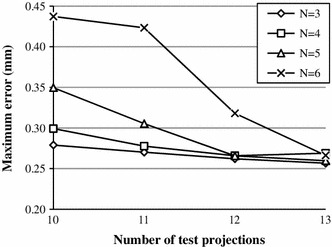
Maximum error as a function of the number of projections, $\sigma_{\text{peak}} = 0.05\,{\text{mm}}, 10^{6}$ simulations

However, the results in Table 1 should be considered in relation to the accuracy of results in Fig. 11, showing the variation of the maximum error as a function of the number of projections.

Two aspects can be observed. Firstly, although the algorithm robustness may be already high, the maximum error may be significantly reduced by increasing the number of projections, and this is particularly evident for $N > 3$ markers. Secondly, in all cases the results appear to converge at $n = 13$, leading to the conclusion that using 13 projections is an optimal choice when up to six markers are used.

**Fig. 12:**
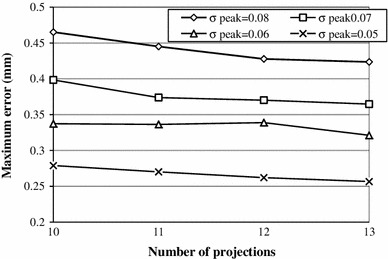
Maximum error in localization of 3 markers for different number of projection directions and$\sigma_{\text{peak}}$

The influence of background noise on the accuracy of peak detection in 1D projections was explored through simulations. It was found that the background noise does influence the accuracy of the sub-pixel peak localization, as illustrated in Fig. 12.

##### *Computational time*

**Fig. 13:**
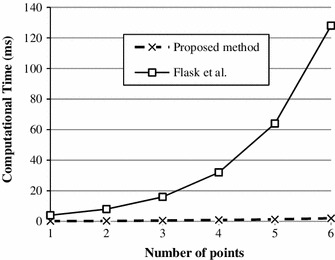
Computational time of the proposed algorithm and the one presented in Flask et al. [1]

Figure 13 shows the computational time in comparison with Flask’s algorithm as a function of the number of markers $N$. The proposed algorithm computes positions of six markers in 1.9 ms while Flask’s algorithm does it in 184 ms.

##### *Targeting accuracy*

Table 2 presents the results of Monte Carlo simulations that were used to analyze the targeting accuracy achieved by using between three and six markers for probe localization. The results correspond to the arrangement suitable for our prostate biopsy probe (Fig. 5). This analysis assumed that the needle and the probe are perfectly made and that there is no deflection of the needle during firing.

**Table 2:** Targeting error using *N* markers

Markers	SD (mm)	Mean error (mm)	Max error (mm)
3	0.111	0.214	0.88
4	0.097	0.191	0.75
5	0.08	0.163	0.57
6	0.07	0.147	0.53

#### Experimental accuracy assessment

##### *Static experiments*

Table 3 shows the analysis of the positional error computed by applying the algorithm to the 20 sets of 1D projections with a marker at eight different locations within the scanner. Standard deviation of the errors was computed for each coordinate as well as for the 3D distance. The standard deviation was lower than 0.06 mm and the maximum error smaller than 0.2 mm for all three coordinates. Similar results for the positional error were obtained in ex vivo experiments.

**Table 3:** Positional error

	x	Y	z	3D
Standard deviation (mm)	0.024	0.040	0.058	0.037
Maximum error (mm)	0.105	0.090	0.148	0.208

**Fig. 14:**
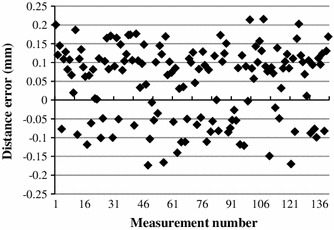
Distance error for a subset of 140 measurements along x. Each point is computed as the difference between distances calculated with marker tracking and those measured with optical encoders

Figure 14 shows a representative subset of the distance errors computed as the difference between distances calculated using marker tracking and those obtained by optical encoders. Error contribution due to an imperfect alignment of the platform with the scanner axes was assumed to be negligible.

The corresponding statistics for independent translations in the x and z directions are presented in Table 4. The average translational error was 0.056 mm while the maximum error was smaller than 0.3 mm. These experiments indicate that sub-millimetre accuracy in tracking can be achieved using the proposed method. It can also be observed that the standard deviation of the distance error is larger than that of the position error shown in Table 3. This is in agreement with the theory which states that, for independent random variables x and y, the variance of their sum or of their difference is the sum of individual variances, (i.e.,$\sigma_{{\left( {X + Y} \right)}}^{2} = \sigma_{{\left( {X - Y} \right)}}^{2} = \sigma_{X}^{2} + \sigma_{Y}^{2}$).

**Table 4:** Distance error statistic: mean error, standard deviation and maximum error

	x translation	z translation
Mean error (mm)	0.049	−0.063
Standard deviation (mm)	0.098	0.168
Maximum error (mm)	0.216	0.283

**Fig. 15:**
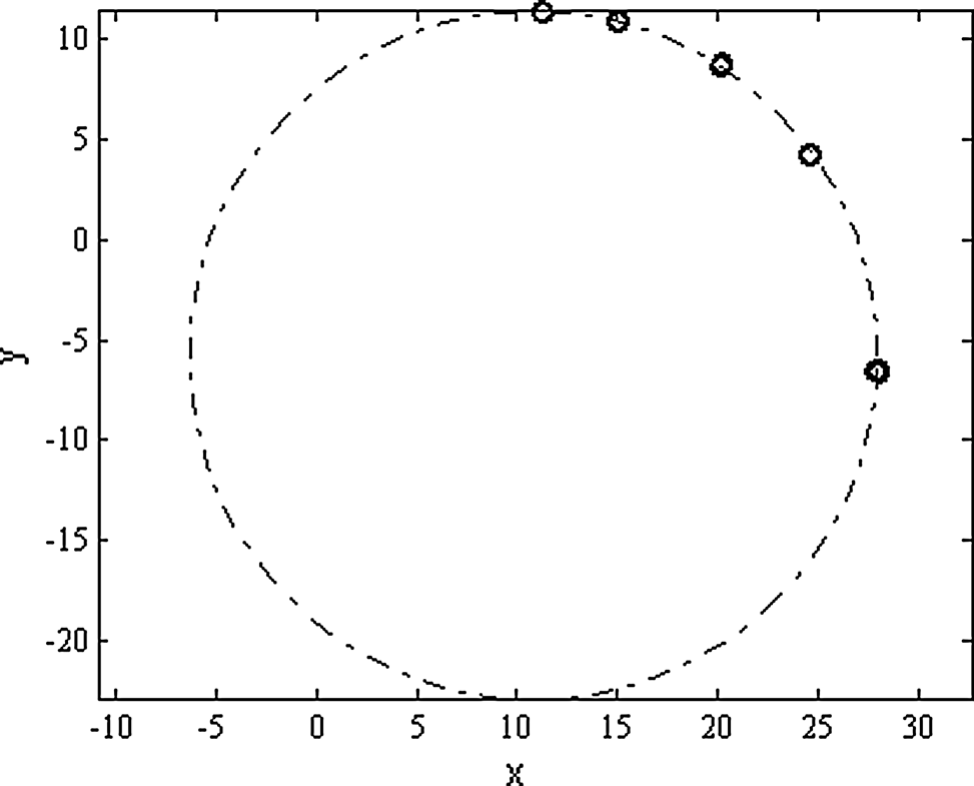
Estimated marker positions and fitted circle. Each position is measured 20 times

Figure 15 illustrates the results of the localization where a fiducial marker was positioned in five different positions (20 times in each) on a circle in the xy plane. High accuracy of localization is proved by computing the goodness of fit of the 100 measured points to the ideal circle *R* ^2^ = 0.9979.

##### *Dynamic experiments*

**Fig. 16:**
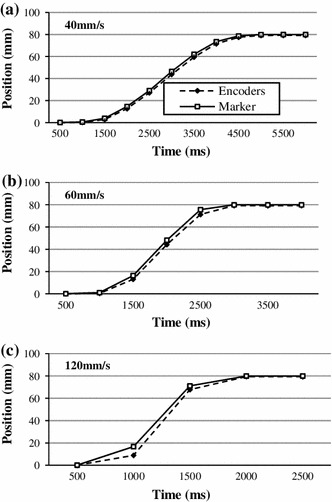
Dynamic tracking at different speeds. Estimated marker positions and measured by encoders

The results of dynamic tests, involving controlled movement of the platform carrying the marker at different speeds are shown in Fig. 16. While the platform was moving, sets of 13 1D projections were repeatedly acquired at regular intervals of 500 ms. The instantaneous position was measured by the encoders after the acquisition of the 6th projection, in order to reduce the time delay between the locations obtained in the two ways.

Table 5 reports the mean and maximum error at the different speeds of the platform. The reported speed is the maximum speed achieved using the s-shaped velocity profile and was controlled by the platform controller. At 40 mm/s the error is lower than 1 mm; at higher speeds, the error increases up to a few mm.

**Table 5:** Distance error for increasing speed

	40 mm/s	60 mm/s	120 mm/s
Mean error (mm)	0.39	1.17	3.07
Max error (mm)	0.72	3.65	7.81

#### Application: MRI guided prostate biopsy

**Fig. 17:**
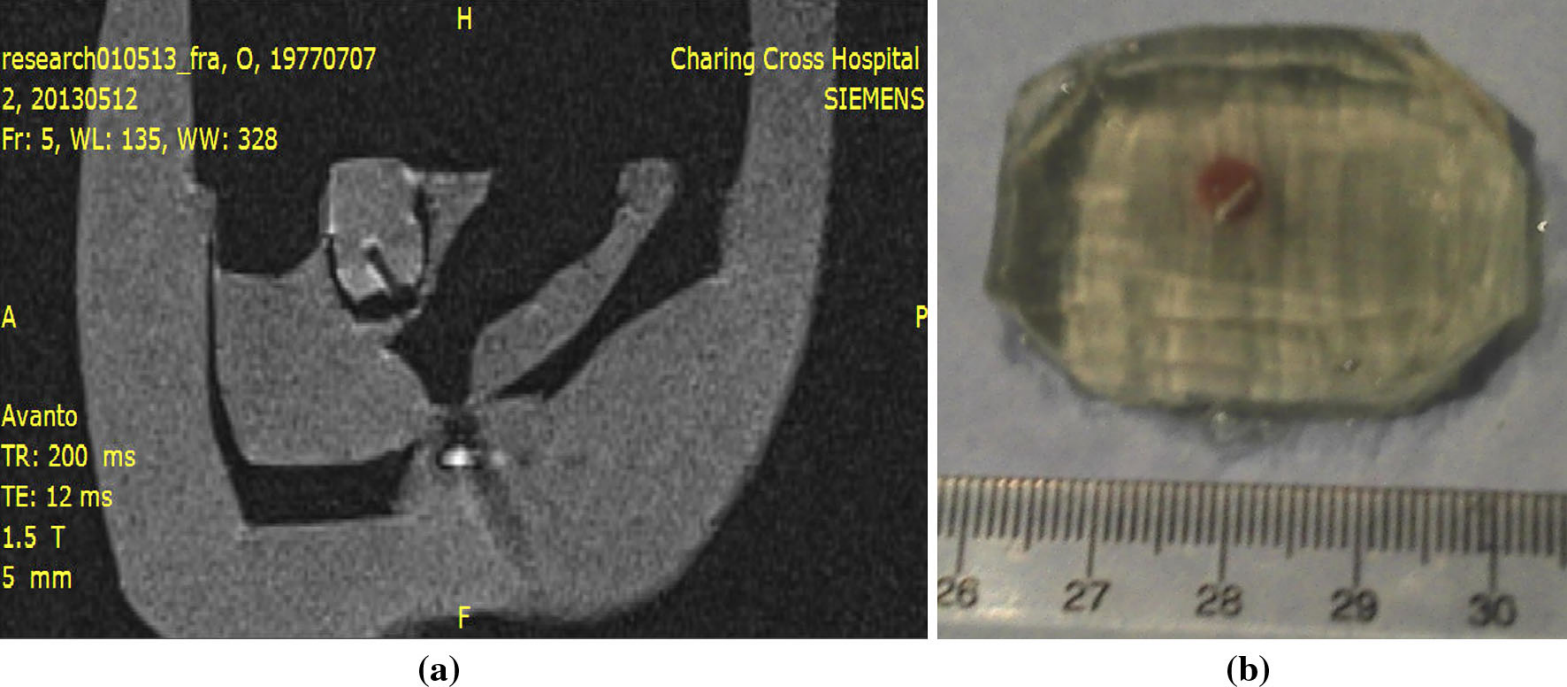
Targeting results: **a** sagittal MR image after firing; **b** photo of the actual target used in the experiment, with the needle track visible

The MRI guided biopsy procedure was carried out in five trials, involving the integrated system and the use of the pelvic model and the prostate phantoms described previously. A trial was considered to be successful if the target sample was acquired, which was evident from its colour, and this was achieved in all trials. Figure 17a presents a sagittal MR image scanned after firing. Figure 17b presents the prostate gland phantom incorporating a 4 mm diameter spherical lesion with the needle track clearly visible.

During targeting, the displayed images of the needle appeared somewhat jittery due to the noise, so a moving average filter that averages five samples was implemented. The stability of the images was improved noticeably, but at the expense of introducing an error due to the delay in the computed positions. Conveniently, this error diminishes as the movement becomes slower in the final phase of the targeting.

#### Volunteer experiments

In all experiments involving volunteers the localization method was found to perform successfully, without failures, i.e., correct peaks were always identified under prolonged operation lasting several minutes. Experiments involving the biopsy probe taped and held tightly between the subject’s legs showed that the SNR of the marker signal was sufficiently high under coil loading and noise conditions similar to that during the intervention. When the markers were taped to the subject’s anterior abdominal wall, where maximum motion artefacts might be expected due to the respiratory motion, the results did not indicate any signal smearing and the localization performed equally successfully over periods of several minutes.

## Discussion

The localization method described here has been shown to be both accurate and fast, while being able to track *N* markers simultaneously.

### Accuracy and robustness

Using a set of 13 pre-defined 1D projections was shown to be optimal in terms of minimizing the localization error and maximizing the robustness, while the penalty in terms of computational time was minimal. The number of projections can be traded against robustness and accuracy for optimized results in a specific situation. Furthermore, by using the *Gaussian* interpolation the accuracy of the position of the peak estimation was significantly improved compared to using the Maximum Pixel Intensity method.

The algorithm is heuristic and two failure modes were identified. First, the algorithm fails if any two markers are positioned too close to each other in relation to the tolerance $\varepsilon$. In practice this problem is unlikely and it may be readily solved by adequately placing the markers on the instrument. The second failure mode is related to a situation where symmetries exhibited by the arrangement of the markers and the chosen directions of the projections generate too many coincident peaks, such that the remaining projections cannot remove all of the fictitious points. This may be solved by choosing a more suitable marker arrangement for the given application or by changing the projection directions in a way that would break the symmetries.

### Determination of the tolerance

Optimizing the value of the tolerance ε is an important but complex problem to address. Too small a value for ε will remove too many candidate points whereas too large *ε* will keep some fictitious points in. Our experiments and simulations indicated that the main factors to be considered when determining the tolerance value are the stochastic nature of the peak position and the identification of peaks that are very close to each other.

By setting the tolerance to ten standard deviations, the stochastic nature was resolved. However, the situation is different when the two peaks are close to each other. It was found that peak merging may occur when peaks are within two pixels from one another and that the position of the identified peak is not affected by merging. In order to accommodate peak merging, the tolerance was automatically enlarged to two pixels size in situations where fewer than *N* peaks are detected. In this way, merged peaks were represented by the identified one. This solution did not affect the accuracy nor robustness of our localization method, since the candidate points are computed by making use only of the projections that do have *N* distinct peaks.

It is well known [31] that positional errors may result from resonance offset errors, such as those when the markers are in a region of an inhomogeneous field near the edges of the imaging volume, or in regions with magnetic distortions caused by differences in magnetic susceptibility. This positional error may affect the robustness of our method, because in the test projections it may change the distance between a projected candidate point and its corresponding peak. If this causes the tolerance to be exceeded, then some of the correct points may be wrongly removed. One way to remedy this problem is to increase the tolerance.

### Application in MRI-guided interventional procedures

The proposed method may be applied to localize either semi-active or active markers, when only one receiver channel is used. However, we have focused on the use of semi-active markers, as it simplifies the instrument design, manufacturing and testing, and avoids cabling issues and the associated safety hazards [32]. We have experienced no significant heating at the relatively low RF exposure levels used in our experiments. The dimensions and the flexibility of use of the semi-active markers make them particularly suitable for MRI-guided interventions involving small devices.

It was observed that, when a human subject is present in the scanner, the peak amplitude of the marker signal may decrease, but it was still sufficiently high for the peak detection method to function correctly and consistently. In practice, obtaining a sufficiently strong signal from the markers can always be expected to be a concern, so the interventional system should be designed such that this is not compromised. Marker orientation to the main field is a key aspect and it is preferable to adopt a manipulator configuration that ensures that markers are always kept perpendicular to B_0_, as was the case in the prostate biopsy system.

The dynamic tracking test proved the reliability of the method in the presence of motion, with accuracy depending on the speed. For the anticipated speeds of tool movement in interventional procedures, the marker positional error was estimated to be within 1 mm.

In preclinical trials the targeting time for prostate biopsy was found to be of the order of 5 min. The proposed prostate biopsy MR-guided method was considered by the clinical staff to offer significant advantages in terms of being able to target previously identified lesions, avoiding the need for extensive random sampling and increasing the accuracy of the biopsies.

Targeting errors such as those due to mechanical deflection of the needle during firing have not been assessed and are potentially significant. Under these conditions, pre-clinical trials were conducted to investigate the ability to successfully sample small targets, of the size specified by the clinician as being clinically relevant. Although these trials were successful, a detailed accuracy of the interventional system needs to be carried out as part of future work, in order to fully assess the errors and influence of factors such as needle firing speed and tissue properties.

In the proposed prostate biopsy procedure, the anatomical MR images are only acquired near the start of the intervention, just before the targeting begins, and immediately before a possible needle release. In the latter case the images are used for the verification of the targeting before the needle is released. Both stages involve obtaining multiple images across the volume of interest, in which the clinician recognizes and identifies the target lesion. In this situation the main benefit of the high update rate of the probe tracking is an improved control of the probe position. In other applications it may be required to update specific MR images simultaneously with tracking, so the faster localization method will help improve the overall image acquisition speed.

Preclinical trials have also revealed that the noise in the system may result in a noticeable jitter in the estimated position of the probe, which may prove distracting to the clinician, especially when performing fine adjustments of the manipulator during targeting. This was easily overcome by implementing a simple moving average filter, resulting in a much steadier display. As a consequence of the fast sampling time (10 Hz), averaging of the last five positions and the slow movement of the probe in the final phase of a targeting, the lag introduced by filtering was not noticeable and it did not impede operator’s actions in any way. The use of the Kalman filter would be an optimal solution for this problem and it will be considered in the next stages of system development.

## Conclusion

A method for localization of *N* markers in 3D based on a novel algorithm for processing 1D projections was presented and proved to be considerably faster than previously proposed methods. Computational time for up to six markers required less than 2 ms. High accuracy was achieved by using optimal reference projections to compute candidate points and by applying Gaussian interpolation in peak detection. An update rate of 10 Hz was achieved with localization error lower than 0.3 mm. The reliability of the method when markers move while performing an intervention was demonstrated and resulted in maximum error 0.7 mm for a speed anticipated by interventional procedures. Experimental targeting error was estimated to be about 1 mm and, we predict a reduction of up to two times by employing up to six markers. An important aspect of the manipulator design is that its remote-centre mechanism maintains the orientation of the markers perpendicular to the main field in all positions, maximizing the peak amplitudes during tracking. Accurate marker localization leads to accurate accurate and consistent targeting in MR guided interventions, while the speed of the method enables high frame-rate display that is comfortable for the clinician and enhances the overall performance.
